# Evaluation of 6LoWPAN Generic Header Compression in the Context of a RPL Network

**DOI:** 10.3390/s24010073

**Published:** 2023-12-22

**Authors:** Thibaut Vandervelden, Diana Deac, Roald Van Glabbeek, Ruben De Smet, An Braeken, Kris Steenhaut

**Affiliations:** 1 Department of Engineering Technology (INDI), Vrije Universiteit Brussel, Pleinlaan 2, 1050 Brussels, Belgium; thvdveld@vub.be (T.V.); roald.van.glabbeek@vub.be (R.V.G.); an.braeken@vub.be (A.B.); 2 Department of Electronics and Informatics (ETRO), Vrije Universiteit Brussel, Pleinlaan 2, 1050 Brussels, Belgium; diana.deac@vub.be (D.D.); rubedesm@vub.be (R.D.S.); 3 Communications Department, Technical University of Cluj-Napoca, Memorandumului 28, 400114 Cluj-Napoca, Romania

**Keywords:** 6LoWPAN, Generic Header Compression, Internet of Things, Low-power Wireless Personal Area Networks, Modified and Improved Header Compression

## Abstract

The Internet of Things (IoT) facilitates the integration of diverse devices, leading to the formation of networks such as Low-power Wireless Personal Area Networks (LoWPANs). These networks have inherent constraints that make header and payload compression an attractive solution to optimise communication. In this work, we evaluate the performance of Generic Header Compression (6LoWPAN-GHC), defined in RFC 7400, for IEEE 802.15.4-based networks running the IPv6 Routing Protocol for Low-Power and Lossy Networks (RPL). Through simulation and real-device experiments, we study the impact of 6LoWPAN-GHC on energy consumption and delays and investigate for which scenarios 6LoWPAN-GHC is beneficial. We show that all RPL control packets are compressible by 6LoWPAN-GHC, which reduces their transmission delay and as such their vulnerability to interference. However, for the devices under study transmitting at 250 kbit/s, the energy gain obtained from sending a compressed packet is outweighed by the energy needed to compress it. The use of 6LoWPAN-GHC causes an energy increase of between 2% and 26%, depending on the RPL packet type. When the range is more important than the bandwidth and a sub-GHz band is used at 10 kbit/s, an energy gain of 11% to 29% can be obtained, depending on the type of RPL control packet.

## 1. Introduction

The Internet of Things (IoT) allows ubiquitous devices to interconnect and communicate seamlessly. The networks formed by these devices, Low-powerWireless Personal Area Networks (LoWPANs), have unique characteristics. These networks should operate with minimal power consumption to ensure a long battery life for the connected devices. They are designed for short-to-medium-range communication and have a limited bandwidth compared to other wireless technologies. Moreover, they are susceptible to interference. LoWPAN devices have a limited processing power and memory, which can be a constraint when implementing more complex IoT applications.

Enabling Internet Protocol version 6 (IPv6) in these resource-constrained networks provides access to IPv6’s advantages. Some of these are a simplified header structure, a significantly larger address space and seamless connectivity. Often, the IEEE 802.15.4 standard [1]. is selected as the Medium Access Control (MAC) and physical layer in LoWPANs, for which the maximum frame size is 127 bytes. After adding the link-layer security and frame overhead, only 81 bytes are available for data coming from upper layers. As the standard IPv6 header is already 40 bytes, not much transport and application data can be carried in one frame. Considering all this, the header compression of IPv6 packets is very important.

The 6LoWPAN Internet Engineering Task Force (IETF) working group was formed in 2004 to define a compression format for IPv6 to be used in LoWPANs. In 2007, the working group defined IPv6 over Low-power Wireless Personal Area Networks (6LoWPANs) in RFC 4944 [2], amended by RFC 6282 [3] in 2011. These proposed standards define compression mechanisms for IPv6 headers, extension headers and User Datagram Protocol (UDP) headers as well as a fragmentation mechanism for IPv6 packets. Compression formats for other protocols, such as the Transmission Control Protocol (TCP) or Internet Control Message Protocol (ICMP), are not defined in these RFCs.

In this article, we focus on Generic Header Compression (6LoWPAN-GHC), defined in RFC 7400 [4], which is an extension of Next Header Compression (6LoWPAN-NHC), defined in RFC 6282. The goal of 6LoWPAN-GHC is to define a compression format that any protocol can use and as such avoid the need for specific formats for every header. 6LoWPAN-GHC is based on LZ77 compression [5], making use of a dictionary and so-called bytecodes, where bytecodes are byte-size decompression instructions.

Our work contributes to this domain by implementing 6LoWPAN-GHC in the Rust programming language and by evaluating the implementation for wireless sensor networks that use the IPv6 Routing Protocol for Low-Power and Lossy Networks (RPL) as the routing protocol. This evaluation, through a simulation study and experiments on a real testbed, will highlight when it is advantageous to use 6LoWPAN-GHC by considering performance parameters, such as the compression ratio, energy consumption and delay.

Because of their structure, RPL control packets have a good chance of being compressed with the 6LoWPAN-GHC mechanism. Indeed, depending on the type, they contain one or more IPv6 addresses and consecutive bytes of zeros. The IPv6 addresses have a high probability of being matched with the dictionary, and consecutive bytes of zeros will be replaced by a single decompression instruction. To summarise, the contributions of our work are the following:

- An implementation of 6LoWPAN-GHC in the Rust programming language on top of the smoltcp [6] library;
- An evaluation of the implementation by considering the following performance parameters: the compression ratio, energy consumption and delay;
- Insights into the conditions under which 6LoWPAN-GHC could be efficient.

This work is structured as follows: After a related work section, in which different compression techniques are discussed, Section 3 gives an introduction to the 6LoWPAN and RPL landscapes. Section 4 explains how 6LoWPAN-GHC functions. In Section 5, we define the performance parameters used for the evaluation of 6LoWPAN-GHC. When to use 6LoWPAN-GHC is further discussed in Section 6, with Section 7 concluding this article.

## 2. Related Work

Modified and Improved Header Compression (MIHC) [7] relies on the fact that many IPv6 packets sent by a given node are usually transmitted to the same destination, meaning that many packets contain the same information in their headers. The core concept is to remove redundant fields in IPv6 headers of correlated packets. To do so, the first packet of a sender node contains an uncompressed IPv6 header. A receiver node will generate a Unique Pair Identifier (UPI), a random and unique 8-bit number, and will store the IPv6 header along with the UPI in a table that links them. Afterwards, the receiver node will transmit the UPI to the sender node. Subsequent packets transmitted by that sender node to the same receiver node will use the UPI instead of the full IPv6 header. This approach increases the throughput significantly, mainly for bigger packets that would otherwise need fragmentation.

In recent years, the IETF worked on Static Context Header Compression (SCHC), a replacement for 6LoWPAN that was discussed by Gomez et al. [8]. It relies on a static context shared between the sender and receiver, simplifying the compression process. Compression rules known by each node in the network define the operation. When transmitting a packet, the compressor selects the appropriate rule to compress the header, replacing it with an identifier. The standard also defines a fragmentation mechanism, to split larger packets into fragments that fit within the maximum frame size. The SCHC fragmentation is specifically designed for Low-Power Wide Area Networks (LPWANs)’ star topology and very small payload sizes. Moreover, SCHC offers reliability by including the No-ACK, ACK-always and ACK-on-Error modes. The benefits of using SCHC have also been studied by Moons et al. [9]. They compared the overhead of the SCHC compression and fragmentation mechanism with 6LoWPAN compression, showing that it is the only protocol that allows the use of end-to-end IPv6 connectivity on devices using SigFox, LoRa and DASH-7.

One limitation of the SCHC protocol is that the context is static and must be distributed before deployment. Moons et al. [10] introduce two context registration mechanisms for SCHC networks. The first mechanism extends the SCHC protocol with an adaptation layer, which is responsible for context registration. The selection of the appropriate action is determined based on specific rules and analysis of incoming packets. Another approach for managing SCHC context configuration involves using the Internet Control Message Protocol for IPv6 (ICMPv6) control messages. An SCHC Control Message is defined to facilitate the configuration of rules for both communication ends. The authors show that the proposed solutions for context registration can lead to a reduced energy consumption and enable resource-constrained devices to self-register and establish their context with the corresponding network gateway.

Robust Header Compression (ROHC) is another compression technique designed to reduce the overhead of network and transport layer headers. It was developed by Jonsson et al. [11] to address the challenges in scenarios with a limited bandwidth and lossy links. ROHC achieves compression by identifying and encoding redundancies in packet headers. Initially, packets are sent uncompressed, but after the redundancy identification and encoding, only the difference between their headers is sent. A context is kept by both the compressor and decompressor. ROHC can handle packet loss and packet corruption by including a mechanism that ensures that the decompression remains reliable. Although ROHC is a powerful header compression technique, it can be challenging to implement it on resource-constrained devices, because ROHC requires a significant computational effort.

Table 1 summarises the compression formats discussed in this section. There are other compression techniques for protocols in the context of 6LoWPAN. O’Flynn [12] wrote a draft proposal for a compression format for ICMPv6 headers. However, it was never finished. In [13,14], the authors define compression techniques for Internet Protocol Security (IPsec) headers and the Datagram Transport Layer Security (DTLS) headers and payload, respectively. In [14], 6LoWPAN-GHC is used for compressing DTLS encrypted data, whilst a new compression format is defined for the various DTLS headers. No in-depth performance analysis has been conducted for this compression format. Geetha et al. [15] propose a technique for compressing IPv6 addresses. This technique is also applicable for SCHC.

Considering that the described compression techniques are either too complex to implement on constrained devices, require a shared context or are too protocol specific, we will focus on the benefits of using 6LoWPAN-GHC. By reducing the size of the packets, 6LoWPAN-GHC will reduce the transmission delay and ensure a lower energy consumption during packet transmission. Nevertheless, compressing will consume energy. Thus, the compression mechanism of 6LoWPAN-GHC qualifies as energy-efficient if the energy saved during transmission outweighs the energy spent on compression, one of the aspects investigated in this article.

To the best of our knowledge, no former studies on the performance of 6LoWPAN-GHC have been published, which is surprising for a technique that is already 9 years old. We decided to investigate its performance in the context of a RPL network.

## 3. Preliminaries

**Figure 1:**
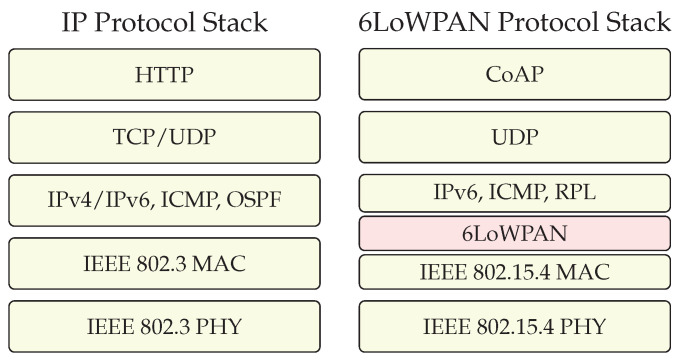
Example of Internet Protocol (IP) over IEEE 802.3 (PHY and MAC) [16] (**left**) and IPv6 over Low-power Wireless Personal Area Network (6LoWPAN) over IEEE 802.15.4 (PHY and MAC) [1] (**right**) protocol stacks.

6LoWPAN is a protocol that enables the use of IPv6 in LoWPANs. LoWPANs connect to other IPv6 networks via edge routers, which manage network interconnection. The IPv6 protocol stack in 6LoWPAN resembles a standard Internet Protocol (IP) stack, with some dissimilarities (Figure 1). At the level of the physical and MAC layers, the IP stack can run over protocols such as IEEE 802.3 (Ethernet) [16] and IEEE 802.11 (Wi-Fi) [17]. Conversely, 6LoWPAN operates over low-power wireless links and often uses IEEE 802.15.4 as the underlying physical and MAC layer. Moreover, the 6LoWPAN stack exclusively supports IPv6 for network layer communication and uses an adaptation layer. In a IP stack, both Internet Protocol version 4 (IPv4) and IPv6 can be used as internetworking protocols. They both count on the help of ICMP for, amongst other things, error reporting, network diagnostics and neighbour discovery [18]. In the IP stack, routing protocols, such as Open Shortest Path First (OSPF), populate the forwarding tables needed by the routers to guide IP packets in the right direction. These classic link state-based routing protocols create too much communication overhead for LoWPANs and are replaced by lightweight distance vector protocols such as the RPL, a routing protocol optimised for routing from node to sink.

In the 6LoWPAN stack, the common choice for a transport protocol is the lightweight UDP. This protocol can undergo compression. At the application layer of the IP protocol stack, the Hypertext Transfer Protocol (HTTP) is frequently used, running over the TCP. Following a client–server model, the HTTP involves clients initiating requests to servers. For the 6LoWPAN stack, the lightweight Constrained Application Protocol (CoAP) is the preferred option and enables a constrained sensor node to take the role of server. The CoAP serves a purpose similar to the HTTP but is specifically designed for constrained devices and low-power networks. Application gateways can easily translate between HTTP and CoAP requests and responses.

The 6LoWPAN adaptation layer fulfils three tasks: (1) compressing headers, (2) fragmenting compressed packets to fit in the Maximum Transmission Unit (MTU) of IEEE 802.15.4, and (3) forwarding them. It is important to specify that 6LoWPAN only defines the format of forwarding headers and not a forwarding mechanism.

### 3.1. Header Compression

Header compression minimises IPv6 header fields using common and contextual information. In RFC 4944 [2], a simple header compression mechanism is defined, IPv6 Header Compression (6LoWPAN-HC). This compression method is used for compressing the IPv6 header and the headers following the IPv6 headers. The IPv6 headers are compressed with IPv6 Header Compression 1 (6LoWPAN-HC1), whereas the headers following the IPv6 header are compressed with IPv6 Header Compression 2 (6LoWPAN-HC2).

With RFC 6282 [3], 6LoWPAN-HC1 and 6LoWPAN-HC2 were completely replaced with IP Header Compression (6LoWPAN-IPHC) and 6LoWPAN-NHC, respectively. From now on, when mentioning 6LoWPAN compression we refer to 6LoWPAN-IPHC and 6LoWPAN-NHC.

The main idea behind 6LoWPAN-IPHC is to exploit the fact that many fields in the IPv6 header remain constant or exhibit predictable patterns in certain communication scenarios. 6LoWPAN-IPHC operates by defining rules and contexts that help devices to identify which fields can be elided and which ones must be included in the compressed header.

**Figure 2:**
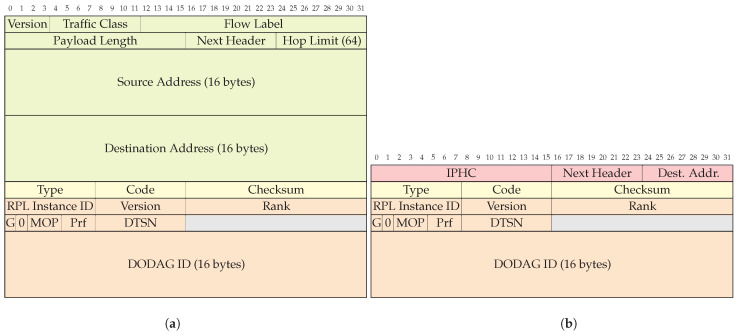
Example of how an IPv6 Routing Protocol for Low-Power and Lossy Networks (RPL) DODAG Information Object (DIO) packet is compressed by IPv6 over Low-power Wireless Personal Area Network (6LoWPAN). (**a**) The uncompressed packet. (**b**) The 6LoWPAN compressed packet. Note that only the Internet Protocol version 6 (IPv6) header is compressed since no compression format is defined for Internet Control Message Protocol for IPv6 (ICMPv6). The source IPv6 address is fully elided, and the destination IPv6 address can be shortened since it is a multicast address. The traffic class and flow label are elided since they are not used in this case. The payload length is not needed and is removed as well. Since the hop limit is 64, it can be elided as well.

An example of how 6LoWPAN compresses an ICMPv6 packet is shown in Figure 2. Figure 2a shows the structure of the packet before compression, and Figure 2b shows the structure after compression. In this example, the traffic class and flow label are elided since they were set to zero and not used. The payload length is removed because it is something that can be calculated from the bytes following the 6LoWPAN-IPHC header. The hop limit has a value of 64, which is a common value and is represented with only 2 bits in the 6LoWPAN-IPHC header. The IPv6 source address is elided because it can be reconstructed using the source link-layer address of the originator of the message. Finally, the IPv6 destination address is a multicast address, which in this case can be reduced to only 1 byte. 6LoWPAN-NHC is not defined for ICMPv6 packets; therefore, the next header field is kept in the compressed packet. The example shows that the IPv6 header was reduced from 40 to 4 bytes.

### 3.2. Fragmentation and Reassembly

Through fragmentation and reassembly, defined in RFC 4944, IPv6 packets are split into smaller frames to meet MTU requirements and are reassembled at the destination to reconstruct the original packet. Each fragment is assigned a number that helps in reordering and reassembling the fragments.

### 3.3. Layer-Two Forwarding

Two forwarding mechanisms are possible in 6LoWPANs: mesh-under and route-over. Mesh-under forwarding, also named layer-two forwarding, occurs at the link layer. The adaptation layer manages the mesh-under forwarding by making forwarding decisions based on the 6LoWPAN headers. Even though RFC 4944 defines the format of mesh frames, it does not specify a mesh routing protocol to fill in the forwarding table. An example of such a protocol is Ad hoc On-Demand Distance Vector Routing (AODV) [19]. For route-over forwarding, the forwarding decisions are made at the level of the network layer. An example of such a routing protocol, defined by the IETF, is the RPL.

The RPL [20] organises the network in a specific tree structure called a Destination Oriented Directed Acyclic Graph (DODAG). The network construction is triggered by the root node with the help of DODAG Information Object (DIO) messages, which carry information about the characteristics of the DODAG. Nodes that receive these messages and want to be part of the network might answer with a Destination Advertisement Object (DAO) message, to which they can obtain a DAO Acknowledgement (DAO-ACK) as a response. The DIO messages are periodically sent by the devices that are part of the network for maintenance purposes. The DODAG Information Solicitation (DIS) messages are sent by nodes that want to join an already formed network.

The RPL control messages, DIO, DAO, DAO-ACK and DIS, are ICMPv6 messages. They are carried in the Content field of an ICMPv6 packet (Figure 2a).

## 4. Generic Header Compression

One of the main problems of 6LoWPAN-NHC is that it does not define any compression for headers other than UDP or IPv6 extension headers. 6LoWPAN-GHC, defined in RFC 7400 [4], tries to solve this issue. It defines a compression mechanism that can compress any header or header-like payload. 6LoWPAN-GHC uses the same principles as the LZ77 compression format [5]. It has a dictionary, which can be referenced to achieve compression.

The RFC 7400 document defines three additional 6LoWPAN-NHC dispatch values. These values are used to indicate that UDP, ICMP and IPv6 extension headers are compressed using the 6LoWPAN-GHC format. For the UDP, the header is compressed using the 6LoWPAN-NHC compression format and only the UDP payload is compressed using 6LoWPAN-GHC.

### 4.1. Decompression

6LoWPAN-GHC decompression is based on a dictionary and bytecodes. Bytecodes are byte-sized instructions for the decompressor. Before processing the compressed data, a dictionary is initialised with the source and destination IPv6 addresses of the packet. The dictionary is also appended with 16 bytes, for which the values are given in the standard (here shown as hexadecimal values): 16 fe fd 17 fe fd 00 01 00 00 00 00 00 01 00 00. The values 16 fe fd and 17 fe fd are used in DTLS handshake and application packets, respectively, with fe fd being the DTLS version. Furthermore, two variables *sa* and *na* are initialised to 0 and used for performing extended backreferencing. After the initialisation of the dictionary and variables, the decompressor starts reading the compressed bytes one by one. The compressed bytes contain bytecode instructions. These instructions can perform five different actions:

1. Copy compressed bytes to the decompression buffer. The bytecode contains the number of bytes to copy to the decompression buffer, with a maximum of 96 bytes.
2. Append zeros to the decompression buffer, with a maximum of 17 zeros. The number of zeros is contained in the bytecode.
3. Set up *sa* and *na* to some specified values, contained in the bytecode. These variables are used for the backreferencing.
4. Perform backreferencing using the *sa* and *na* variables and extra information contained in the bytecode. The backreferencing is performed using the already decompressed bytes, prepended with the dictionary.
5. A stop instruction is only used when compressing IPv6 extension headers.

### 4.2. Compression

**Figure 3:**
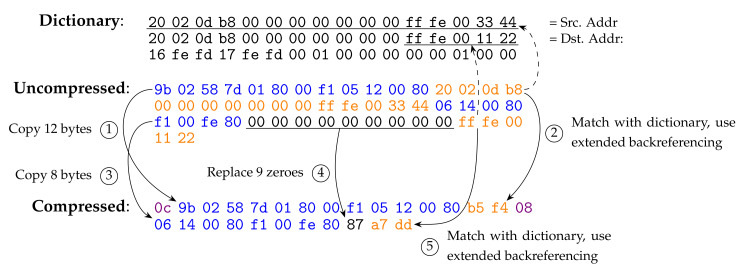
An example of how an ICMPv6 RPL DAO packet is compressed using Generic Header Compression (6LoWPAN-GHC).

How to compress is not defined in the standard, but the chosen compression algorithm should generate a compressed payload that conforms to the decompression rules. By leaving the choice or design of the compression algorithm open, it can be adapted and tuned to specific use cases. To better explain how a compression algorithm can operate, an example is provided in Figure 3. First, a buffer is initialised with the dictionary, as described in Section 4.1. We iterate over the uncompressed packet and calculate the number of consecutive zeros. We also look for a match between the uncompressed data and the dictionary. When there are no consecutive zeros present and no match is found, the data are just copied into the compression buffer, as depicted in Figure 3 with steps 1 and 3. The copied data are prepended with the copy instruction, which contains the number of bytes to be copied when decompressing.

When a match is found, the backreferencing instruction is used. If the length of the match is higher than 6 or the match is more than 6 bytes to the left, then the extended backreferencing instruction needs to be used. This is shown in Figure 3 with steps 2 and 5. Step 2 completely elided the source address and step 5 found a partial match with the destination address. When a long sequence of zeros is found, it can be elided using the zero instruction, as shown in step 4, where nine zeros are replaced by only one instruction.

In case of IPv6 extension headers, a stop instruction will be appended. This instruction is needed to show where the compression of each intermediate header ends. This is not needed for the upper-layer headers, since they always appear at the end of the packet.

## 5. Methodology and Measurements

Our implementation of 6LoWPAN-GHC was added to the smoltcp [6] library (the implementation is not merged into upstream smoltcp but can be found here: https://gitlab.com/etrovub/smartnets/smoltcp-rpl-ghc, (accessed on 21 August 2023)). This library is written in the Rust programming language [21]. Rust is a compiled programming language that can be used for embedded devices. It has the advantage of being a memory-safe programming language, as opposed to C. At the same time, Rust can maintain the same performance as C. The smoltcp library already contains common Internet protocols, such as UDP, TCP and ICMPv6. It also contains an implementation of 6LoWPAN-IPHC, 6LoWPAN-NHC and the fragmentation mechanism from RFC 4944 [2].

To evaluate our implementation, we simulate a network of nodes running the RPL. RPL control packets contain fields that are unused and set to zero and fields that contain IPv6 addresses. Thus, using 6LoWPAN-GHC can be beneficial because the IPv6 addresses might be found or partially matched in the dictionary, and consecutive zeros are replaced with the zero instruction. For example, in a DAO packet, two of the fields, Reserved and Flags, are set to zero and the DODAGID field is an IPv6 address.

In addition to the simulation study, we run our implementation on real nodes, using nRF52840 devices [22]. These devices are based on the 32-bit ARM Cortex-M4 architecture, running at a clock frequency of 64 MHz. They have 1 MB of flash memory and 256 KB of Random Access Memory (RAM) and house a 2.4 GHz transceiver, with IEEE 802.15.4 protocol support. This is a typical current generation device used in 6LoWPANs, concerning its clock speed, power draw and system-on-chip design. To run smoltcp on them, the Embassy [23] framework is used. Embassy provides Hardware Abstraction Layers (HALs) for various embedded devices and uses smoltcp as its networking stack.

To assess the efficiency of 6LoWPAN-GHC, we consider three different performance parameters. The first parameter is the compression ratio, which measures the ratio of the original packet size to the compressed one (Equation (1)). A higher compression ratio indicates a more efficient compression.

(1) $$\mathrm{compression}\;\mathrm{ratio}=\frac{\mathrm{uncompressed}\;\mathrm{packet}\;\mathrm{size}}{\mathrm{compressed}\;\mathrm{packet}\;\mathrm{size}}$$

The second parameter is the energy consumption. An outgoing packet travels through the network stack, where it is built and compressed. We denote the energy for building the IPv6 packet as $E_{b}$ and the energy for compressing the packet as $E_{c}$. After building and compressing the packet, it is processed and transmitted by the radio unit. The transmission energy is denoted as $E_{tx}$. The power consumption of an active microcontroller is denoted by $P_{mcu}$, and the transmission power consumption is denoted by $P_{tx}$. They are hardware-dependent and can be found in the datasheet of devices. The total energy (*E*) required for processing and transmitting a packet can be defined as follows:

(2) $$\begin{array}{cc} E & =E_{b}+E_{c}+E_{tx}\\ \; & =P_{mcu}\cdot \left(t_{b}+t_{c}\right)+P_{tx}\cdot t_{tx} \end{array}$$

For compression without 6LoWPAN-GHC, $t_{c}$ contains the 6LoWPAN-IPHC compression time and the 6LoWPAN-NHC compression time. We denote the non-6LoWPAN-GHC compression time by $t_{c,nhc}$. When using 6LoWPAN-GHC, $t_{c}$ contains the 6LoWPAN-IPHC compression time and the 6LoWPAN-GHC compression time. This is denoted by $t_{c,ghc}$. The transmission energy depends on the compression mechanism used since it modifies the number of bytes that are transmitted. We denote the transmission energy for a 6LoWPAN-GHC compressed packet by $E_{tx,ghc}$ and the transmission energy for a non-6LoWPAN-GHC compressed packet by $E_{tx,nhc}$.

The energy consumed in each step, from building to transmitting a packet, is dependent on the efficiency of the implementation and on the hardware used. Usually, the power usage of a microcontroller ($P_{mcu}$) is lower than the power required by the radio when transmitting ($P_{tx}$). Note that compression reduces the time of transmitting a packet and thus reduces the $E_{tx}$. However, a 6LoWPAN-GHC compression attempt may take quite some processing time without yielding enough gain in reducing the packet size. Compression might be impossible or hard and slow. Performing the 6LoWPAN-GHC compression is only beneficial from an energy perspective when

(3) $$E_{c,ghc}-E_{c,nhc}<E_{tx,nhc}-E_{tx,ghc}$$

The last parameter that we consider is the delay. In telecommunication systems, multiple causes of delay can be identified, such as processing, queuing, access, transmission and propagation. Processing and transmission delays are impacted by the compression mechanism. Together, they influence queuing delays. The transmission delay is defined as the packet size divided by the data rate. Compression attempts to decrease the packet size and transmission delay but increases the processing delay. To decrease the total delay, the following equation should be satisfied:

(4) $$d_{c,ghc}-d_{c,nhc}<d_{tx,nhc}-d_{tx,ghc}$$

The variable $d_{tx,ghc}$ represents the transmission delay with 6LoWPAN-GHC compression, whereas $d_{tx,nhc}$ represents the transmission delay without 6LoWPAN-GHC compression. The 6LoWPAN-GHC processing delay is denoted by $d_{c,ghc}$, whereas $d_{c,nhc}$ represents the processing delay for 6LoWPAN-NHC.

### 5.1. Evaluation by Means of a Simulator

A basic simulator was developed by our research group in the context of the evaluation of the smoltcp library. A node in the simulator has a message queue for incoming frames and one for outgoing frames. Nodes have a predefined transmission range. When a node wants to transmit a frame, it puts the frame into its outgoing queue. The simulator then iterates over all these queues and puts the frames in the incoming queues of the correct receivers, only when the receiver is in transmission range of the sender. In that case, the simulator assumes a perfect frame delivery, not taking into account physical layer issues, such as interference, collision, etc.

**Figure 4:**
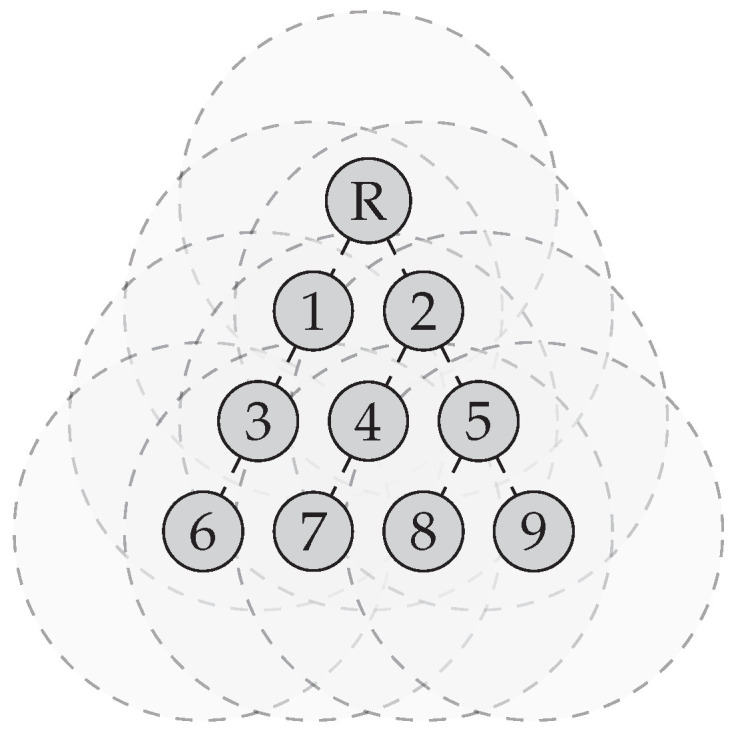
Example of an RPL network in the simulator. Node R is the root of the RPL network. The grey dashed circles represent the transmission ranges of the nodes.

The simulator evaluates the 6LoWPAN-GHC implementation by simulating 10 nodes, with 1 root node and 9 normal nodes. The root node will initiate and maintain the network organisation with the help of RPL control messages. The topology formed by the RPL is shown in Figure 4.

**Figure 5:**
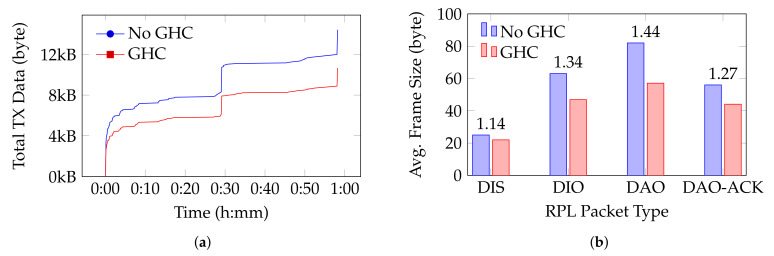
Comparison of network performance considering the number of bytes transmitted over the network and the average frame size in a RPL network with and without 6LoWPAN-GHC. Over the period of 1 h, 7 DODAG Information Solicitation (DIS), 100 DODAG Information Object (DIO), 60 DAO and 60 DAO Acknowledgement (DAO-ACK) frames were captured. (**a**) The total number of bytes sent over the network in 1 h. The RPL topology of this network is depicted in Figure 4. (**b**) Average frame size for each RPL packet type captured over a period of 1 h, with and without 6LoWPAN-GHC. The compression ratio is shown above the bars.

To evaluate the implementation of 6LoWPAN-GHC, we capture frames exchanged by the nodes during a 1-h simulation. We run this simulation once without and once with 6LoWPAN-GHC. The information collected during the 1-h simulation is shown in Figure 5. Figure 5a shows the total number of bytes transmitted for both scenarios. The sudden increase in control traffic after 30 min of simulation time is due to the renewal of the RPL routes. This renewal happens every 30 min. We can see that the total number of bytes sent over time is less when using 6LoWPAN-GHC. Transmitting fewer bytes means consuming less bandwidth, potentially reducing congestion.

Relevant details about the RPL control packets exchanged during a 1-h simulation are shown in Figure 5b. A DAO control packet has the highest compression ratio, being 1.44. In the simulation, the DAO packet contained the RPL Target option and the Transit Information option. These options contain an IPv6 address or a prefix, which makes it likely that they match with parts of the dictionary and thus are compressible. The DAO packet also contains the DODAG ID, which matches partially with the dictionary. Unfortunately, the DAO contains an IPv6 Hop-by-Hop extension header, which was not compressible since it did not have a match with the dictionary, nor did it have any zeros that could potentially have been compressed.

DAO-ACKs have a compression ratio of 1.27. Even though the DODAG ID in the DAO-ACK can be compressed, the IPv6 Hop-by-Hop extension header cannot. The compression ratio for a DIO in our simulation is 1.34 since the DODAG ID was compressible. A DIS, an ICMPv6 packet where the content is two consecutive bytes (the Flags and Reserved fields), has a compression ratio of 1.14, where the zero bytes are represented by one bytecode. DIOs and DISs are multicast messages and their content usually remains unchanged during the lifetime of the network.

When using MIHC [7] compression, where a shared context is used between a sender and a receiver node, a DIO packet would result in a total frame size of 60 bytes. This frame contains 15 bytes for the IEEE 802.15.4 frame header, 1 byte for the unique pair identifier and 44 bytes for the RPL DIO packet. Thus, the compression ratio is 1.05, compared to 1.34 when using 6LoWPAN-GHC. Computing the compression ratio of a RPL packet when using SCHC or ROHC is more difficult as these compression mechanisms are less intuitive to apply to our scenario. For SCHC, a specific rule could be created for compressing RPL packets, which might result in high compression ratios.

Note that the simulation study only considers RPL control traffic, which is shown here to be compressible. Data packets will usually be less compressible as they contain data that match less with the content of the dictionary. This will negatively affect the global compression ratio.

### 5.2. Evaluation on a Real Testbed

The purpose of conducting measurements on a real testbed is to quantify the impact of 6LoWPAN-GHC on energy consumption for handling incoming and outgoing packets. We again ran two sets of measurements: one with 6LoWPAN-GHC enabled and the other without 6LoWPAN-GHC. We measured the power draw of two nRF52840 devices running the RPL protocol. One device served as a RPL root node and the other as a normal node. The power was measured using a Keysight N6705B DC Power Analyzer [24] for a duration of 15 s. During this period, all four types of RPL control messages were sent by the devices. The sampling frequency during the measurements was set to the smallest resolution that the measurement device was capable of, which was 40.96 μs. The accuracy for the current measurements is 8 nA, and for the voltage it is 50 μV. When measuring the power draw, the radio communication was captured using an nRF52840-based radio sniffer, such that the packets could be identified in the power trace. Measurements show that the power draw of the active microcontroller ($P_{mcu}$) is 88 mW, whereas the transmission power draw ($P_{tx}$) is 157 mW.

**Figure 6:**
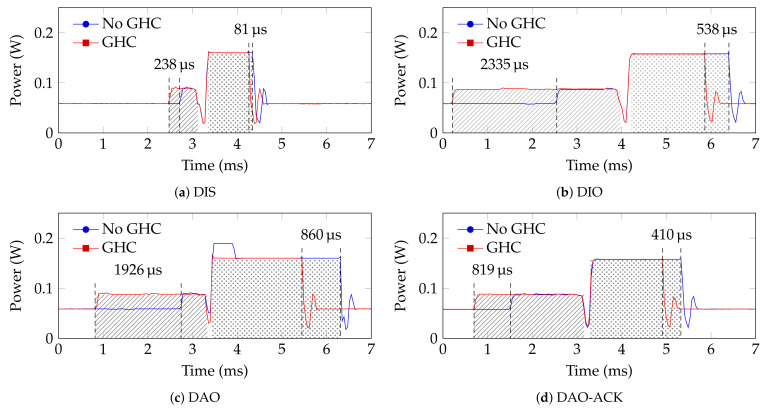
Power measurements over time of a device processing and transmitting different RPL control packets, with and without 6LoWPAN-GHC compression. The hatched area is associated with the processing period of the packet, and the dotted area is associated with the transmission period.

We consider the compression algorithm to be energy-efficient when the energy gained by transmitting fewer bytes is higher than the energy lost in compressing the packet, as shown in Equation (3). This parameter can be computed for each type of RPL control packet. Figure 6 shows the power measurements over time for each type. Before the packet is processed, the microcontroller is in a low-power state. When the microcontroller starts processing and constructing the packet, its power consumption increases. The next increase in power is due to the transmission of the packet. Finally, if the microcontroller has nothing to process, it enters the low-power state. It can be seen that the time taken to process and transmit a control packet depends on its type. The DIS, being the smallest packet, has the lowest processing and transmission time. Applying 6LoWPAN-GHC increased the processing time with 238 μs and decreased the transmission time with 81 μs. In terms of energy, the processing energy increased by 20.94 μJ (+44%). This number reflects the energy spent in compressing the packet with the 6LoWPAN-GHC mechanism, $E_{c,ghc}$ in Equation (3). The transmission energy was reduced by 12.96 μJ (−9%). This is the difference between the $E_{tx,nhc}$ and $E_{tx,ghc}$. Overall, the total energy consumption increased by 8.0 µJ (+4%) for a DIS packet.

For a DIO, which has the highest compression ratio, the energy consumption for the packet compressed increased by $E_{c,ghc}=205.5$ μJ (+164%) and the energy consumption for transmitting that packet decreased by $E_{tx,nhc}\;-\;E_{tx,ghc}=84.95$ μJ (−25%). The total energy consumption increased by 118.2 μJ (+26%). For a DAO and a DAO-ACK, the total energy consumption increased by 34.5 μJ (+7%) and 7.7 μJ (+2%), respectively. It can be observed that none of the RPL control packets satisfy Equation (3).

## 6. Discussion

The simulation study demonstrates relatively high compression ratios for RPL control messages. As a result, the transmitted packets are smaller, which translates into a reduction in the transmission delay. A shorter transmission delay can lead to more traffic accommodation and an increased overall network throughput. It can also reduce the probability of collisions and interference with other devices sharing the same frequency spectrum, which is important in crowded wireless environments.

The testbed performance measurements show that the total energy consumption increased for all RPL control packets. The same applies to the total delay. This increase is caused by the execution of the compression algorithm. Improving the implementation of the compression algorithm might decrease the delay and energy consumption. A possible approach is using a faster dictionary-matching algorithm, such as an Aho–Corasick-based algorithm [25]. The content of the dictionary could be modified, but this does not respect the standard. For example, the last 16 bytes of the dictionary are based on heuristics and might not help in compressing RPL packets. Finding better values for the 16 bytes or removing them from the dictionary might improve the performance of the compression algorithm. A specialised compression format for ICMPv6 can yield better results than a generic one. However, this was not the goal of 6LoWPAN-GHC as this would require a new RFC for every new standard or for every modification of an existing standard.

All measurements in Section 5 are performed for an IEEE 802.15.4-based network, operating on the 2.4 GHz Industrial, Scientific and Medical (ISM) band with a data rate of 250 kbit/s. However, the IEEE 802.15.4 standard also defines lower data rates for transmissions on the 868 MHz band: 10 kbit/s, 40 kbit/s and 100 kbit/s. Table 2 and Table 3 show the calculated impact on the delay and energy consumption for other data rates, respectively. For each data rate, the top bar represents the total delay/total energy for when 6LoWPAN-GHC is used, with the bottom bar being the total delay/total energy for non-6LoWPAN-GHC compressed packets. It is important to highlight that the compression delays/energies remain unchanged for the different data rates. The data rates only affect the transmission delay and transmission energy consumption. Table 2 shows that, on average, for data rates lower than 100 kbit/s, the total delay reduces for all the 6LoWPAN-GHC compressed RPL control packets, satisfying Equation (4). This means that the compression delay introduced by the 6LoWPAN-GHC mechanism does not nullify the decrease in transmission delay achieved by transmitting less data. Once a high enough data rate is used, the impact of the compression delay is noticeable and increases the total delay.

The same conclusion can be drawn for the energy consumption (shown in Table 3). For data rates lower than 100 kbit/s, the total energy consumption is reduced for all the 6LoWPAN-GHC compressed RPL control packets, satisfying Equation (3). The energy increase needed to perform the 6LoWPAN-GHC compression is less than the energy gain obtained by transmitting less data. For data rates equal to or higher than 100 kbit/s, the energy spent performing the 6LoWPAN-GHC compression becomes high enough to have a negative impact on the total energy consumption. If we take, for example, the CC1200 transceiver, which can operate on the 868 MHz frequency band, the transmission power varies between 119 mW and 152 mW. For a DIO, transmitted at 10 kbit/s with the highest transmission power, the total energy consumption is equal to 7.79 mJ with 128 μJ for processing the packet and 7.66 mJ for transmitting the packet. When using 6LoWPAN-GHC, the total energy consumption would be equal to 6.05 mJ, with 332 μJ for compression and processing and 5.72 mJ for transmission. At a data rate of 10 kbit/s, the total energy consumption is reduced by 1.74 mJ (−22%) because of the compression. For a data rate of 40 kbit/s, the total reduction is 0.28 mJ (−14%). At a data rate of 100 kbit/s, there is no change in the total energy consumption. The transmission energy reduction is nullified by the energy spent in 6LoWPAN-GHC compressing.

Note that the results obtained pertain specifically to the energy consumption and transmission delay associated with frame transmission. We observed through measurements that 6LoWPAN-GHC has a negligible impact on the decompression and processing of received packets. Furthermore, the energy consumed for creating the dictionary is negligible compared to the energy spent for compressing the packet. Creating the dictionary is a simple copy operation of the IPv6 addresses from the packet.

## 7. Conclusions

In this article, we investigate the impact of 6LoWPAN-GHC on various performance parameters for RPL-based networks. We implemented 6LoWPAN-GHC in the Rust programming language, a high-performance compiled language that can be used on embedded devices. The implementation was added on top of the smoltcp library.

By means of simulation, we show that 6LoWPAN-GHC reduces the total number of transmitted bytes, with compression ratios between 1.14 and 1.44 for different RPL control packets. Reducing the frame size reduces the transmission delay. This is beneficial from a networking point of view. Shorter packets reduce the probability of collisions and interference with other devices sharing the same frequency spectrum. The overall network throughput could also increase as the channel could be accessed more frequently.

The IEEE 802.15.4 standard defines multiple data rates: 10 kbit/s, 40 kbit/s, 100 kbit/s and 250 kbit/s. Lowering the data rates allows for an increased transmission range, with the cost of an increased transmission delay. For lower data rates, a 6LoWPAN-GHC compressed control packet experiences an up to 19% decrease in the total delay compared to a non-6LoWPAN-GHC compressed packet. For data rates higher than 100 kbit/s, the total delay experienced by a 6LoWPAN-GHC compressed packet is larger than the total delay experienced by a non-6LoWPAN-GHC compressed packet. This is due to the fact that the transmission delay at a high data rate is already much shorter and so is its reduction thanks to compression. This reduction is nullified by the extra processing delay, caused by executing the compression algorithm.

A similar conclusion can be drawn for total energy consumption. For lower data rates, e.g., 10 kbit/s, it is reduced by up to 22% for a DIO packet. For DISs, DAOs and DAO-ACKs, the energy is reduced by up to 11%, 29% and 20%, respectively. Measurements on real testbeds show that the total energy consumption per 6LoWPAN-GHC compressed control packet for a device under study using the 2.4 GHz ISM band with a data rate of 250 kbit/s is larger than the total energy consumption per non-6LoWPAN-GHC compressed packet. The additional energy spent on performing 6LoWPAN-GHC compression on RPL control packets is higher than the gain in energy obtained by reducing the transmission time.

Note that our conclusions are in the context of a RPL network, where the control packets are compressible with 6LoWPAN-GHC due to their structure. For data packets, the compression ratio could be smaller, meaning that the total energy consumption and total delay could only increase.

In future work, it would be interesting to investigate the impact of the static bytes in the dictionary. In the case of the RPL, they do not seem to have an impact on the compression ratio. We anticipate that having a larger dictionary actually increases the compression delay since there are more bytes to match against the data.

## Figures and Tables

**Table 1:** Contextualisation of compression formats for IPv6 packets.

Compression Format	Fragm.	Link Type	Compression
ROHC [11]	**✗**	Any	The compressor and decompressor are implemented as state machines. A shared context is maintained by both the compressor and decompressor using feedback messages, which modify the state machines of both ends. Redundancy in packet headers that can be inferred from previously transmitted packets is removed. **Only headers are compressed, not the payload**.
SCHC [9]	**✓**	LPWAN	Compression is defined in terms of fields and a shared static context. The shared context is a set of rules that define how to compress and decompress fields. When both ends of the connection know the shared context, they can compress and decompress **any header or payload** as defined by the rules.
6LoWPAN [2,3]	**✓**	LPWAN	Compression is defined for **IPv6 headers, IPv6 extension headers and UDP headers**. Compression and decompression can be stateless or stateful. In the stateful case, address contexts are shared. **6LoWPAN-GHC** [4] is an extension for 6LoWPAN that allows the compression of **any header or payload** not handled by 6LoWPAN.
MIHC [7]	**✓** ^1^	LPWAN	**Only the IPv6 header** is compressed. The first IPv6 header is transmitted uncompressed. Subsequent packets transmitted by the same sender and receiver use a Unique Pair Identifier (UPI) instead of the full IPv6 header. The UPI is only 8 bits long and identifies the IPv6 header.

^1^ It is not clear from the paper whether fragmentation is supported. We assume it uses the fragmentation mechanism as defined by 6LoWPAN.

**Table 2:** Total delay for RPL control packets for different data rates. For each row, the total delay for a 6LoWPAN-GHC compressed packet is shown in the top bar, with the bottom bar being for non-6LoWPAN-GHC compressed packets. The 6LoWPAN-GHC compression reduces the transmission delay for lower data rates.

		6LoWPAN-GHC Proc. delay 6LoWPAN-GHC Transm. delay
		6LoWPAN Proc. delay 6LoWPAN Transm. delay
**Packet Type**	**Data Rate (kbit/s)**	**Total Delay (Processing + Transmission Delay)**
DIS	10	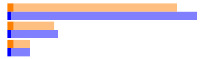
	40	
	100	
DIO	10	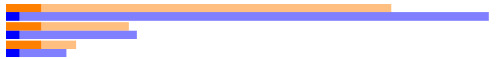
	40	
	100	
DAO	10	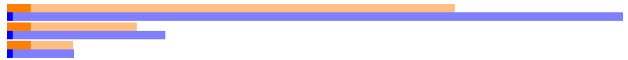
	40	
	100	
DAO-ACK	10	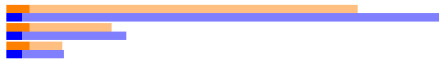
	40	
	100	
		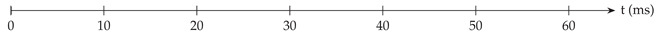

**Table 3:** Total energy consumption for RPL control packets for different data rates. For each row, the total energy consumption for a 6LoWPAN-GHC compressed packet is shown in the top bar, with the bottom bar being for non-6LoWPAN-GHC compressed packets. The 6LoWPAN-GHC compression reduces the transmission energy for lower data rates.

		6LoWPAN-GHC Proc. delay 6LoWPAN-GHC Transm. delay
		6LoWPAN Proc. delay 6LoWPAN Transm. delay
**Packet Type**	**Data Rate (kbit/s)**	**Total Energy Consumption (Processing + Transmission Energy)**
DIS	10	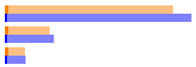
	40	
	100	
DIO	10	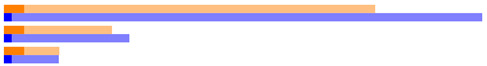
	40	
	100	
DAO	10	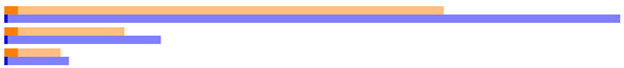
	40	
	100	
DAO-ACK	10	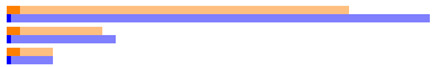
	40	
	100	
		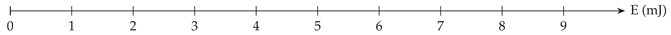

## Data Availability

The data that support the findings of this study are available from the corresponding author, T.V., upon reasonable request.

## Abbreviations

The following abbreviations are used in this manuscript:

**6LoWPAN**	IPv6 over Low-power Wireless Personal Area Network
**6LoWPAN-GHC**	Generic Header Compression
**6LoWPAN-HC**	IPv6 Header Compression
**6LoWPAN-HC1**	IPv6 Header Compression 1
**6LoWPAN-HC2**	IPv6 Header Compression 2
**6LoWPAN-IPHC**	IP Header Compression
**6LoWPAN-NHC**	Next Header Compression
**AODV**	Ad Hoc On-Demand Distance Vector Routing
**CoAP**	Constrained Application Protocol
**DAO**	Destination Advertisement Object
**DAO-ACK**	DAO Acknowledgement
**DIO**	DODAG Information Object
**DIS**	DODAG Information Solicitation
**DODAG**	Destination Oriented Directed Acyclic Graph
**DTLS**	Datagram Transport Layer Security
**HAL**	Hardware Abstraction Layer
**HTTP**	Hypertext Transfer Protocol
**ICMP**	Internet Control Message Protocol
**ICMPv6**	Internet Control Message Protocol for IPv6
**IETF**	Internet Engineering Task Force
**IoT**	Internet of Things
**IP**	Internet Protocol
**IPsec**	Internet Protocol Security
**IPv4**	Internet Protocol version 4
**IPv6**	Internet Protocol version 6
**ISM**	Industrial, Scientific and Medical
**LoWPAN**	Low-power Wireless Personal Area Network
**LPWAN**	Low-power Wide Area Network
**MAC**	Medium Access Control
**MIHC**	Modified and Improved Header Compression
**MTU**	Maximum Transmission Unit
**OSPF**	Open Shortest Path First
**RAM**	Random Access Memory
**ROHC**	Robust Header Compression
**RPL IPv6**	Routing Protocol for Low-Power and Lossy Networks
**SCHC**	Static Context Header Compression
**TCP**	Transmission Control Protocol
**UDP**	User Datagram Protocol
**UPI**	Unique Pair Identifier
